# Transcriptomic Signature of Frailty in Older Patients With Cardiovascular Disease Undergoing Cardiac Surgery or TAVI

**DOI:** 10.1002/jcsm.13846

**Published:** 2025-06-05

**Authors:** Omar Baritello, Simon H. Sündermann, Kristian Espinosa‐Garnica, Jörg Kempfert, Markus Jähnert, Nick L. Beetz, Dominik Geisel, Jasmin Gaugel, Julia Rominger, Ursula Müller‐Werdan, Catrin Herpich, Kristina Norman, Alexandra Chadt, Hadi Al‐Hasani, Heinz Völler, Annett Salzwedel, Heike Vogel

**Affiliations:** ^1^ Department of Rehabilitation Medicine, Faculty of Health Sciences Brandenburg University of Potsdam Potsdam Germany; ^2^ Research Group Molecular and Clinical Life Science of Metabolic Diseases, Faculty of Health Sciences Brandenburg University of Potsdam Potsdam Germany; ^3^ Department of Cardiothoracic and Vascular Surgery Deutsches Herzzentrum der Charité Berlin Germany; ^4^ Charité‐Universitätsmedizin Berlin Berlin Germany; ^5^ German Center for Cardiovascular Research (DZHK), Partner Site Berlin Berlin Germany; ^6^ German Center for Diabetes Research (DZD e.V.), München‐Neuherberg Munich Germany; ^7^ Department of Experimental Diabetology German Institute of Human Nutrition Potsdam‐Rehbruecke Nuthetal Germany; ^8^ Department of Radiology Charité‐Universitätsmedizin Berlin, Corporate Member of Freie Universität Berlin and Humboldt‐Universität zu Berlin Berlin Germany; ^9^ Berlin Institute of Health at Charité‐Universitätsmedizin Berlin BIH Biomedical Innovation Academy, Germany Berlin Institute of Health Berlin Germany; ^10^ Research Group Nutrigenomics of Obesity German Institute of Human Nutrition Potsdam‐Rehbruecke Nuthetal Germany; ^11^ Department of Geriatrics and Medical Gerontology Charité‐Universitätsmedizin Berlin, Corporate Member of Freie Universität Berlin and Humboldt‐Universität zu Berlin Berlin Germany; ^12^ Evangelisches Geriatriezentrum Berlin gGmbH Berlin Germany; ^13^ Department of Nutrition and Gerontology German Institute of Human Nutrition Potsdam‐Rehbrücke Nuthetal Germany; ^14^ Institute of Nutritional Science University of Potsdam Nuthetal Germany; ^15^ German Center for Cardiovascular Research (DZHK), Partner Site Berlin Berlin Germany; ^16^ Institute for Clinical Biochemistry and Pathobiochemistry, German Diabetes Center (DDZ), Medical Faculty Heinrich Heine University Duesseldorf Germany

**Keywords:** cardiovascular disease, frailty, muscle function, older cardiac patients, sarcopenia, transcriptomics

## Abstract

**Background:**

Deterioration of functional capacity mostly determinates frailty in older patients with cardiovascular disease (CVD). Elucidating the pathophysiological mechanisms of physical frailty is an important goal for improving functional health‐related outcomes. Our objective was the determination of the transcriptomic signature of physical frailty phenotypes in older patients undergoing cardiac surgery.

**Methods:**

Patients aged ≥ 70 years, referred for elective cardiac surgery (e.g., coronary artery bypass graft) or transcatheter aortic valve implantation (TAVI) were recruited. At hospital admission, frailty was assessed based on moderately/severely impaired mobility (Timed Up and Go; ≥ 10 and ≥ 20 s), low gait speed (5‐m Walk Test; ≥ 6 s) or reduced handgrip strength (male ≤ 27, female ≤ 16 kg). Muscle specimens (*M. quadriceps femoris*) were collected during surgery/intervention and used for total RNA isolation and sequencing. Differential gene expression analysis was performed using DESeq2 and regression analyses investigated association between frailty and gene expression levels.

**Results:**

Sixty‐three patients (77.6 ± 4.3 years; 74% male) referred to cardiac surgery (*n* = 34, 54%) or TAVI (*n* = 29, 46%) were included. Overall, 43 patients (70.2%) were characterized as frail by moderately/severely impaired mobility, 19 (30.6%) by low gait speed and 19 (30.2%) by low handgrip strength. In total, 37 patients (59%) experienced ≥ 1 complication (e.g., need of transfusion, atrial fibrillation and delirium); one patient died. Based on transcriptome data, 10 overlapping genes between all physical frailty phenotypes were identified, with *S100A1* showing the strongest differences in expression level (e.g., handgrip *R* ^2^_adj_ = 0.579; *p* = 0.001). Additional functional studies in C2C12 myoblasts demonstrated the impact of S100A1 on muscle function, and a second independent human cohort confirmed that higher S100A1 blood levels were correlated with increased handgrip strength.

**Conclusion:**

Our data highlight the potential role of *S100A1* in the pathophysiological mechanisms of skeletal muscle impairments in older CVD patients and warrants further consideration as a target gene for physical frailty. These findings also advance our understanding of the genetic and biological factors contributing to frailty, potentially guiding future therapeutic strategies to mitigate its impact on health outcomes.

## 1 Introduction

The World Health Organization (WHO) defines frailty as an age‐related syndrome characterized by multisystemic dysregulation leading to physiological decline and increased vulnerability against every day or acute external stressors [1]. The physiological reserves of frail individuals are impaired, leading to maladaptive responses to stressors (e.g., hospitalization and surgery) with progressive loss of functioning and adverse health‐related outcomes [2], notably in patients with cardiovascular disease (CVD), too [3].

In clinical practice, frailty is mainly assessed according to its key determinants characterizing physical functionality: unintentional weight loss, reduced strength, slow gait speed, exhaustion and low physical activity [4]. Common phenotypes of frailty such as slow gait speed and a lower handgrip strength correlate with a poorer prognosis [5, 6]. Limited mobility, operationalized by the Timed Up and Go (TUG) test combining gait speed, strength, balance and coordination, was found to be a valid predictor of increased mortality after transcatheter aortic valve implantation (TAVI) and surgical procedures in octogenarians [7, 8]. Wider domains of frailty are mapped by patients' cognitive performance [9] and nutritional status [10]. Frailty has a broad intersection with sarcopenia, defined as concomitant loss of muscle mass and strength. Hence, sarcopenia is also considered a subcomponent of frailty [10]. For older patients undergoing TAVI or heart surgery, independent prognostic value of the psoas muscle area has actually been demonstrated based on computed tomography (CT) assessment [11].

The underlying biological mechanisms contributing to the physical phenotypes of frailty are complex and multifactorial [12] including the dysregulation of inflammatory processes, oxidative stress, mitochondrial dysfunction and cellular senescence [13], and it is also influenced by other factors, such as sociodemographic characteristics, psychological conditions, nutritional status, lack of physical activity and comorbidities [13, 14]. However, it still needs to be defined what drives frailty and what are the risk factors for disease progression. Both the environment and genetics influence the dysfunction of the mechanisms associated with frailty. Studies on the genetic determinants of frailty have used diverse methodological approaches, initially focusing on selected candidate single nucleotide polymorphisms (SNPs), shifting to genome‐wide association studies (GWAS) in the previous decade [15]. Recent large‐scale GWAS on frailty have identified several novel susceptibility loci, revealing significant genetic correlations with neuropsychiatric, cardiovascular and inflammation‐related pathways [16, 17]. Biomarker studies have also pointed towards the involvement of various biological pathways in frailty [18, 19, 20]. However, their clinical performance and utility remain limited, leading to the recommendation of using a panel of validated biomarkers instead of single markers to achieve a more precise diagnosis and a deeper understanding of the genetic determinants of frailty and the associated biological pathways remains crucial [17, 20].

Thus, our objective was to improve our understanding of the genetic and biological hallmarks associated with frailty status to set the basis for early detection of the disorder and the development of appropriate treatment strategies. In the present study, older cardiac patients hospitalized for cardiac surgery or TAVI were characterized with respect to frailty phenotypes, and expression profiling of skeletal muscle biopsies as well as bioinformatic data analyses were performed.

## 2 Methods

### 2.1 Study Setting and Patients

**FIGURE 1:**
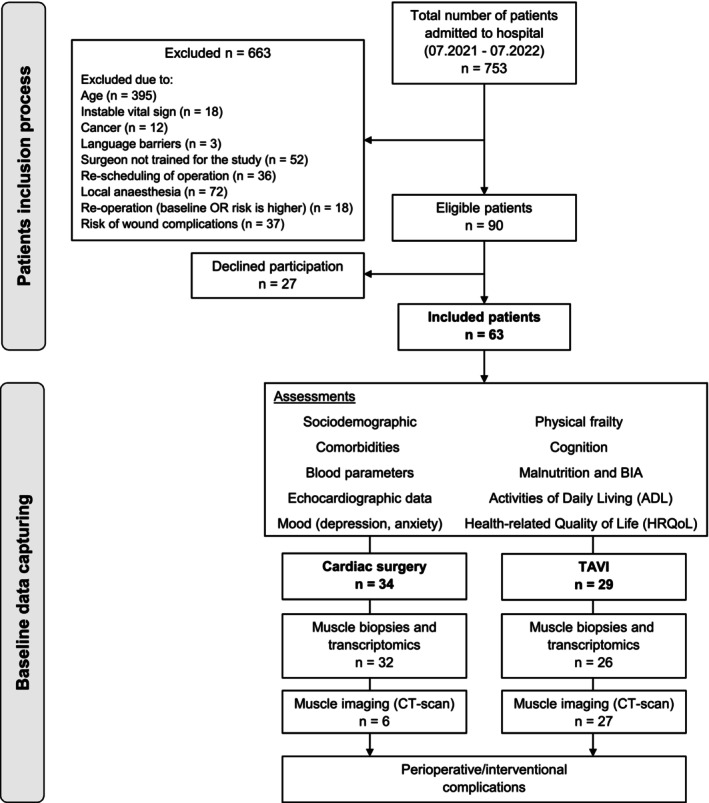
CONSORT flow chart of inclusion process and baseline data capturing. BIA, bioelectrical impedance analysis; CT‐scan, computed tomography scan; TAVI, transcatheter aortic valve implantation.

This prospective monocentric cohort study included 63 patients (≥ 70 years of age) out of 753 older patients admitted to the Department of Cardiovascular Surgery, Charité‐Universitätsmedizin Berlin, for elective cardiac surgery (e.g., coronary artery bypass grafting) or minimally invasive procedure (TAVI) between June 2021 and July 2022 (Figure 1, Supplementary Table S2). The inclusion and exclusion criteria are shown in Figure 1. The study protocol was approved by the Ethics Committee of the Charité‐Universitätsmedizin Berlin (No. EA4/086/21) and registered in the German Clinical Trials Register (DRKS00025449).

### 2.2 Baseline Data Capturing and Clinical Outcome

Sociodemographic data (e.g., age and gender), comorbidities (e.g., diabetes, chronic kidney disease and peripheral artery disease), blood parameters (e.g., serum albumin and haemoglobin) and echocardiographic data (e.g., left and right ventricular ejection fraction) were collected from the medical records of the patients (Figure 1).

The frailty status of the patients with its different components was assessed at hospital admission using a standardized test battery. Patients were classified as frail if presenting a low gait speed (≥ 6 s) in the 5‐Meter Walk Test (5‐mWT) [S1], low handgrip strength measured by hand‐dynamometer (≤ 27 kg male; ≤ 16 kg female) [S2] or an impaired mobility assessed with the TUG test (≥ 10 s moderately and ≥ 20 s severely impaired) [S3]. The validated questionnaires Mini‐Mental State Examination (MMSE) [S5] and the short‐form of the Mini Nutritional Assessment (MNA‐sf) [S6] were administrated for cognitive impairment and for the risk of malnutrition, respectively. The Katz [S7] and the Lawton‐index [S8] questionnaires rated the limitation of basic and instrumental activities of daily living. Health‐related quality of life was evaluated using the Short Form 12 (SF‐12) [S9] while depression and anxiety were monitored using the Patient Health Questionnaire (PHQ‐9) [S10]. The online Society of Thoracic Surgeons adult cardiac surgery risk calculator (STS; version 4.20) and the European System for Cardiac Operative Risk Evaluation (EuroSCORE‐II) were used to estimate the predicted risk of mortality and length of hospital stay (short < 6 days and long > 14 days). The bioelectrical impedance analysis (BIA; Nutrigard‐MS, Data Input, Pöcking, Germany) was used to assess body impedance of the patients (resistance), following a standardized procedure. Resistance values were used to calculate the skeletal muscle mass (SMM) based on the protocol of Otten et al. [S11], and sarcopenic patients were defined according to the criteria of lower handgrip strength (< 27 kg men; < 16 kg women) and lower SMM (< 7 kg/m^2^ men; < 5.5 kg/m^2^ female) [S2].

During surgery or intervention, respectively, the surgeon collected a sample of the *m. quadriceps femoris* (50–100 mg) from each patient through a 1.5‐cm surgical incision at the lateral aspect of the thigh. Fascia was incised along the muscle fibres' direction and a 3 × 3 mm muscle sample was collected. Samples were immediately frozen in liquid nitrogen, stored in a refrigerator (−80°C) and afterwards used for total RNA isolation and sequencing.

Perioperative complications (from surgery/intervention to hospital discharge) defined as a combined endpoint of mortality and major morbidity (e.g., delirium, deep wound infection and atrial fibrillation) [S11, S12] and the postoperative length of hospital stay, measured in days from surgery or procedure to hospital discharge, were documented.

### 2.3 Statistical Analysis

Continuous variables are expressed as means ± standard deviation (SD), and categorical variables as absolute values and percentages. Comparisons between groups were performed using the *t*‐test and the *χ* ^2^‐test, respectively. Association between frailty and perioperative complications were identified using logistic regression models adjusted for age, sex, number of comorbidities and estimated glomerular filtration rate (eGFR) in both the surgery and the TAVI groups. All calculations were performed using the SPSS 29.0 software package (IBM, Chicago, IL, USA).

### 2.4 Gene Expression Analysis in Skeletal Muscle and C2C12 Cells

Total RNA was extracted from 5–10 mg of *m*. *quadriceps femoris* and C2C12 cells using the RNeasy Mini Kit (Qiagen, Hilden, Germany) as per the manufacturer's protocol, with additional DNase treatment and details of transcriptome analysis and quantitative real‐time PCR is provided in the supporting information.

### 2.5 Linkage Analysis, Exercise Training and Data Retrieval From the Human GWAS Catalogue

Genome‐wide linkage analysis of the backcross (NZOxC3H)N2 population was performed as previously described [S16]. Briefly, female NZO mice from our own breeding colonies (NZO/HIBomDife) were mated with male C3H/FeJ (C3H; Helmholtz Center, Munich, Germany) mice to produce F1 hybrids. Male F1 mice were subsequently backcrossed to female NZO to produce N2 mice that were metabolically characterized. Genetic map, genotyping errors and linkage between individual traits and genotypes were assessed with the software package R/qtl (version 1.04‐8) using the expectation maximization (EM) algorithm and 1000 permutations [S17].

Nine‐weeks‐old male C57BL/6J mice (Charles River Germany) were randomly assigned to the exercise (*n* = 24; EX) or sedentary (*n* = 24; SED) group. The endurance training and transcriptomic profiling in skeletal muscle samples was performed as previously described [S18].

Data from human GWAS were obtained from the NHGRI‐EBI GWAS catalogue using FTP servers [S19]. Regional plots for the *S100A1* gene ± 5 kb were generated using R‐version 4.2.3, utilizing a modified version 0.18 of qqman. This adapted version allowed colour coding for variant consequences and replacement of chromosomes with phenotypes. Variant consequences were obtained using the biomaRt R‐package version 2.54.

## 3 Results

### 3.1 Patient Characteristics

Older patients admitted to the Department of Cardiovascular Surgery at the Charité‐Universitätsmedizin Berlin were screened for possible inclusion and 63 participants (≥ 70 years of age) were enrolled in the study: 34 patients (54%) underwent cardiac surgery and 29 (46%) had a TAVI (Figure 1). The mean age of the entire cohort was 77.6 ± 4.3 years, with the TAVI patients being significantly older (Table 1). Forty‐seven patients (74%) were male. There were no significant differences between the surgery and TAVI groups in most of the patient characteristics at baseline. Exceptions to this finding were higher levels of the diagnostic and prognostic biomarkers N‐terminal prohormone of brain natriuretic peptide (NT‐pro‐BNP) and lower concentrations of serum albumin, protein and haemoglobin in TAVI patients. The majority of patients (*n* = 49, 78%) had ≥ 4 comorbidities, while atrial fibrillation was more common in TAVI patients (*n* = 13, 45%), and chronic obstructive pulmonary disease (COPD) was more prevalent in surgical patients (*n* = 11, 32%). Likewise, preprocedural surgery risk scores (STS, EuroSCORE‐II) predicting in‐hospital mortality and length of stay (LoS), which include parameters such as age, sex, comorbidities, medications, blood parameters and surgical factors, were higher in the TAVI population. Before hospitalization, 9 patients were in need of care (care level II or III) and the majority of participants (*n* = 40, 63.5%) lived with a partner or in the family, while 23 patients (34.9%) reported living alone. Both groups reported mean similar limitations (scores < 50) in the physical (PCS) and mental (MCS) health‐related quality of life (SF‐12) and no signs of depression (PHQ‐9 score < 10). In addition, in 33 patients (surgery *n* = 6, TAVI *n* = 27) the area of the psoas muscle (PA, surgery 16.06 ± 3.43; TAVI 16.80 ± 6.86 cm^2^/m^2^) and the PMI (Psoas Muscle Index, surgery 5.52 ± 1.01; TAVI 5.72 ± 2.22) were calculated based on the pre‐interventional CT measurement at the level of third lumbar vertebra.

**TABLE 1:** Patients characteristics.

	Total (*n* = 63)	Surgery (*n* = 34)	TAVI (*n* = 29)	*p*
Sociodemographic				
Age (years)	77.6 ± 4.3	75.2 ± 3.5	80.4 ± 3.5	0.001
Sex (male)	47 (74.6)	25 (73.5)	22 (75.9)	
BMI	26.76 ± 3.43	27.21 ± 3.67	26.23 ± 3.11	0.262
Echocardiographic				
LVEF (%)	52.97 ± 10.40	53.85 ± 9.90	51.93 ± 11.04	0.507
RVEF (%)	54.10 ± 4.63	54.26 ± 4.29	53.90 ± 5.07	0.688
TAPSE (mm)	21.32 ± 5.11	22.21 ± 4.59	20.31 ± 5.56	0.315
PAP (mmHg)	31.13 ± 16.22	28.39 ± 9.69	34.07 ± 20.90	0.173
Blood parameters				
Protein (g/L)	71.08 ± 5.55	72.77 ± 4.31	69.11 ± 6.23	0.027
C‐reactive protein (mg/L)	6.71 ± 17.08	4.99 ± 12.10	8.33 ± 21.17	0.553
Haemoglobin (g/dL)	13.00 ± 1.71	13.61 ± 1.27	12.37 ± 1.95	0.009
HbA1c (%)	6.38 ± 1.01	6.44 ± 1.07	6.30 ± 0.94	0.862
NT‐pro‐BNP (ng/L)	1829.93 ± 2232.81	1296.03 ± 1761.69	2482.48 ± 2586.38	0.019
Serum albumin (g/dL)	4.25 ± 0.39	4.35 ± 0.25	4.11 ± 0.46	0.037
eGFR (mL/min)	59.20 ± 19.36	63.12 ± 17.99	54.93 ± 19.87	0.046
Comorbidities				
High blood pressure	62 (98.4)	33 (97.1)	29 (100)	> 0.999
Renal failure	33 (52.4)	17 (50.0)	16 (55.2)	0.438
Diabetes	31 (49.2)	19 (55.9)	12 (41.4)	0.186
Atrial fibrillation	20 (31.7)	7 (20.6)	13 (44.8)	0.037
PAD	18 (28.6)	8 (23.5)	10 (34.5)	0.248
Malignancy in medical history	15 (23.8)	5 (14.7)	10 (34.5)	0.081
COPD	14 (22.2)	11 (32.4)	3 (10.3)	0.035
Patients ≥ 4 comorbidities	49 (77.8)	26 (76.5)	23 (79.3)	0.667
Risk scores				
STS‐mortality and morbidity (%)	14.39 ± 10.80	10.25 ± 4.43	19.24 ± 13.78	0.001
STS‐LoS—short (%)	35.47 ± 13.10	40.56 ± 12.21	29.54 ± 11.71	0.001
STS‐LoS—long (%)	7.06 ± 6.84	4.95 ± 2.54	9.53 ± 9.21	0.008
EuroSCORE‐II (%)	4.57 ± 6.20	2.63 ± 1.57	6.85 ± 8.49	0.002
Depression and HRQoL				
PHQ‐9 (*n* = 60)	5.7 ± 4.90	5 ± 3.80	6.4 ± 5.80	0.646
SF‐12 (*n* = 59)				
PCS	36.54 ± 6.03	35.11 ± 5.62	38.11 ± 6.18	0.321
MCS	38.77 ± 8.56	38.97 ± 8.36	38.55 ± 8.92	0.549

*Note:* Numbers: mean ± standard deviation or number of patients (%).

Abbreviations: COPD, chronic obstructive pulmonary disease; eGFR, estimated glomerular filtration rate; EuroSCORE‐II, European System for Cardiac Operative Risk Evaluation; HRQoL, health related quality of life; LVEF and RVEF, left and right ventricular ejection fraction; PAD, peripheral artery disease; PAP, pulmonary artery pressure; PCS and MCS, Physical and Mental Component Summary; PHQ‐9, Patient Health Questionnaire; SF‐12, 12‐Item Short‐Form Health Survey; STS, Society of Thoracic Surgeons‐Risk score; STS‐LoS short and long, predicted risk of short (< 6 days) and long (> 14 days) postoperative length of stay; TAPSE, tricuspid annular plane systolic excursion; TAVI, transcatheter aortic valve implantation.

### 3.2 Frailty Parameters

Assessment of frailty was based on a battery of standardized measurements (Table 2). Forty‐three patients (70.2%) were characterized by moderate (≥ 10 s) or severe (≥ 20 s) mobility impairment performed by the TUG, with TAVI patients requiring a longer time to complete the task. Decreased gait speed (≥ 6 s, 5‐mWT) or handgrip strength (≤ 27 kg in men; ≤ 16 kg in women) were detected in 19 patients. According to the MNA‐sf, 10 patients (16%) were at risk of malnutrition (MNA‐sf score < 12) with a mean score of 12.6 ± 2.2 (surgery 12.9 ± 1.7; TAVI 12.3 ± 2.8) in the sample. The cognitive performance of the patients investigated was, on average, slightly reduced (MMSE mean score 26.5 ± 2.7; surgery 26.5 ± 2.9; TAVI 26.5 ± 2.5), with 17 patients (52%) in the surgical and 16 in the TAVI group (55%) characterized by mild to severe cognitive impairment (MMSE score ≤ 27). One male patient was defined as sarcopenic based on low handgrip strength and low SMM.

**TABLE 2:** Characteristics of the physical frailty phenotypes.

Assessments	Total (*n* = 63)	Surgery (*n* = 34)	TAVI (*n* = 29)
TUG (s)	12.72 ± 4.75	10.80 ± 3.37	14.88 ± 5.18*
Moderately impaired mobility (≥ 10 s)	38 (62.3)	18 (54.5)	25 (89.3)
Severely impaired mobility (≥ 20 s)	5 (7.9)	None	5 (17.9)
5‐mWT (s)	5.83 ± 2.34	5.33 ± 1.64	6.40 ± 2.84
Low gait speed (≥ 6 s)	19 (30.6)	8 (24.2)	11 (37.9)
Handgrip (kg)	28.73 ± 8.98	30.45 ± 9.40	26.73 ± 8.17
Low handgrip (≤ 27 kg ♂; ≤ 16 kg ♀)	19 (30.2)	7 (20.6)	12 (41.4)

*Note:* Numbers: mean ± standard deviation or number of patients (%). *Significant difference (*p* < 0.05).

Abbreviations: TAVI, transcatheter aortic valve implantation; TUG, Timed Up and Go test; 5‐mWT, 5‐Meter Walk Test.

**FIGURE 2:**
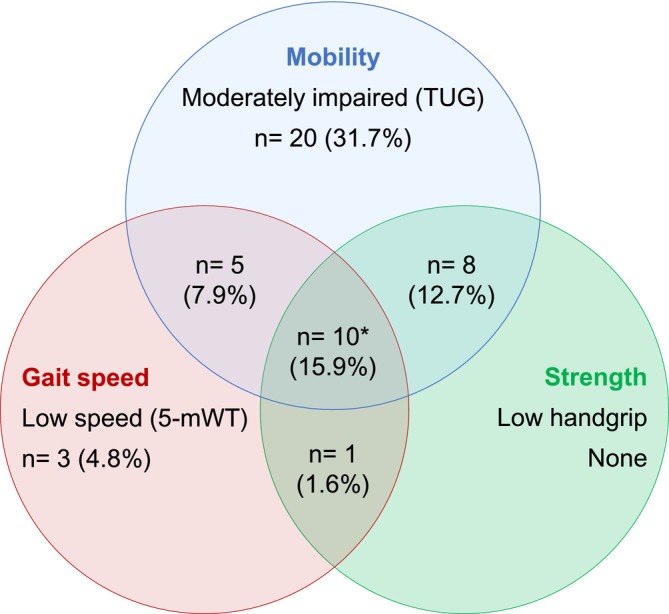
Overview of the study population in terms of physical frailty. Venn diagram representing number, percentage and overlap of patients assigned to the different parameters characterizing physical frailty. *Of the 10 patients in the central intersection, 5 had a severe impaired mobility.

In addition, the potential overlap of physical frailty phenotypes was investigated (Figure 2). We found 14 patients (22.2%) characterized by the presence of two co‐existing phenotypes, mostly with moderate mobility impairment coinciding with low gait speed or low handgrip strength. In 10 patients (15.9%), all three phenotypes of frailty intersected.

### 3.3 Clinical Outcomes

In the periprocedural course, 37 patients (59%) experienced ≥ 1 complication(s) (surgery *n* = 28, 82.4%; TAVI *n* = 9; 31.0%), specifically need of transfusion (*n* = 24, 38%), atrial fibrillation (*n* = 14, 22%), delirium (*n* = 12, 19%), pneumonia (*n* = 8, 13%), renal failure (*n* = 6, 10%), stroke/TIA (n = 2, 3.2%) or intubation > 24 h (*n* = 2, 3.2%); one patient died. Overall, patients were discharged from hospital 9.81 ± 5.11 days after surgery or intervention, with TAVI patients being discharged earlier (8.11 ± 4.81 days) than surgical patients (11.32 ± 4.94 days).

In adjusted regression analyses, longer time in the TUG was significantly associated with a higher risk of complications in the TAVI (odds ratio: OR 1.33 per 1 s, 95% confidence interval: CI 1.02–1.75, *p* = 0.038) and not in the surgery group (OR 1.01, 95% CI 0.75–1.37, *p* = 0.934) (Figure S1). No association was found between complications and 5‐mWT, handgrip, MMSE or MNA‐sf.

### 3.4 Transcriptomic Profiling in Human Skeletal Muscle

**FIGURE 3:**
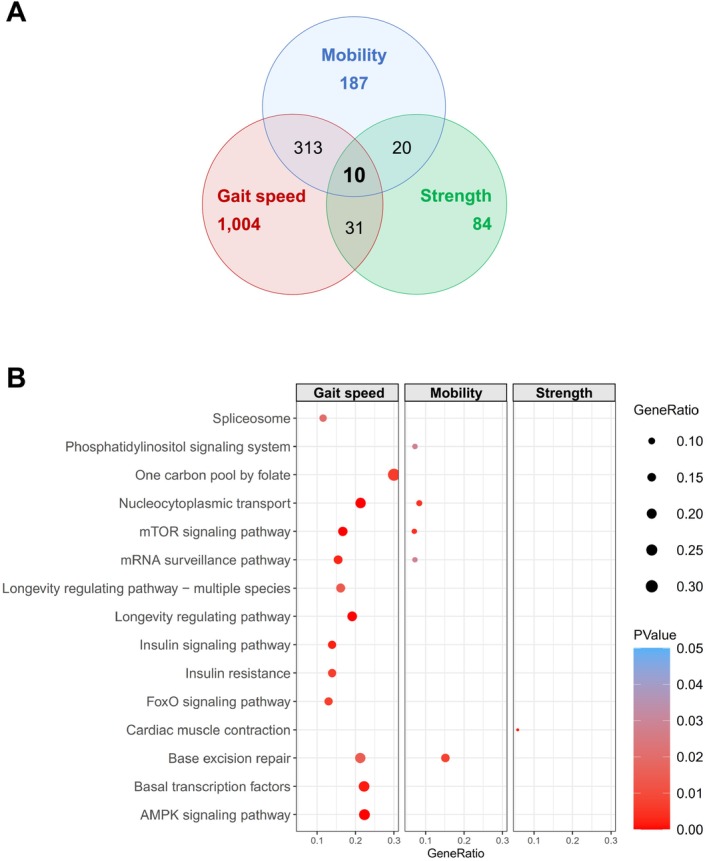
Transcriptome analysis in human muscle biopsies. (A) Venn diagram depicting the number of genes that correlated significantly with one or multiple parameters of physical frailty. (B) Dot plots of KEGG enriched pathway analysis of the top 150 genes associated (*p* < 0.05) with parameters of physical frailty. Colour of the circles indicates *p* value and dot size is proportional to the number of differentially expressed genes (DEG) in the given pathway. 5‐mWT, 5‐Meter Walk Test; TUG, Timed Up and Go test; and handgrip strength.

Although the TAVI and surgery groups differ in certain characteristics, we hypothesize that the pathogenesis of physical frailty observed in both groups is based on similar molecular changes in muscle‐specific processes. Hence, RNA‐sequencing was performed from skeletal muscle biopsies of all patients. By applying the linear model, 1358 genes out of about 24 800 genes were detected with an altered expression associated with a low gait speed (5‐mWT), whereas 530 and 145 genes showed an altered expression with impaired mobility (TUG) and low handgrip strength, respectively (Figure 3A). To investigate whether a similar set of genes is affected in its expression between different parameters of physical frailty, the overlap of the whole transcriptome data was performed. As shown in Figure 3A, 10 genes revealed a significant linear regression for low gait speed, impaired mobility as well as low handgrip strength. The comparative analysis revealed a higher overlap between low gait speed and mobility than for the handgrip parameter, indicating a common genetic basis and regulation.

To evaluate the molecular events and pathways involved in the transition from healthy to frail, Kyoto Encyclopedia of Genes and Genome (KEGG) pathway enrichment analysis was performed. The highest number of enriched metabolic pathways was associated with a low gait speed; several of the differentially expressed genes are involved in pathways known to regulate muscle metabolism and function such as mTOR (mammalian target of rapamycin) signalling, insulin signalling, insulin resistance and AMPK (AMP‐activated protein kinase) signalling and others (Figure 3B). Interestingly, only fivepathways were in common between gait speed and mobility, and no shared pathways between gait speed and handgrip strength (Figure 3B).

### 3.5 Top Genes Displaying Differential Expression in the Skeletal Muscle

**FIGURE 4:**
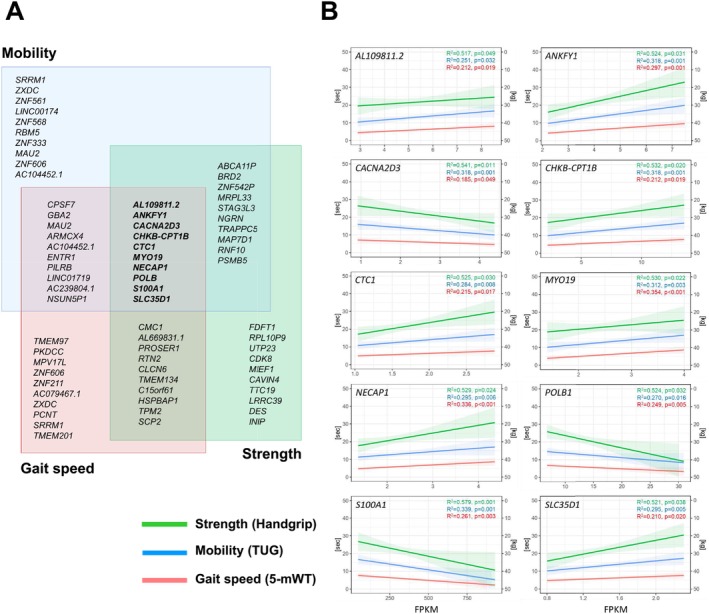
List of genes associated with physical frailty. (A) Venn diagram listing the top 10 genes with an altered expression for the indicated parameter of physical frailty and the corresponding overlaps, ranked by the lowest *p* value. (B) Linear plots representing the correlation between the expression in skeletal muscle with impaired mobility (TUG), low gait speed (5‐mWT) and low handgrip strength of the top 10 overlapping genes affecting physical frailty. FPKM, fragments per kilobase per million mapped fragments.

For an in‐depth analysis, we listed the top 10 genes with a significantly altered gene expression pattern related to low gait speed, impaired mobility and low handgrip strength and the different overlaps, ranked by the lowest *p* value (Figure 4A). For the overlap between the measured parameters of physical frailty, 10 genes including one long non‐coding RNA (lncRNA), one non‐coding RNA (ncRNA) and eight protein‐coding genes could be defined. Linear regression plots for these top genes are exemplarily presented for the parameter impaired mobility in Figure 4B.

**FIGURE 5:**
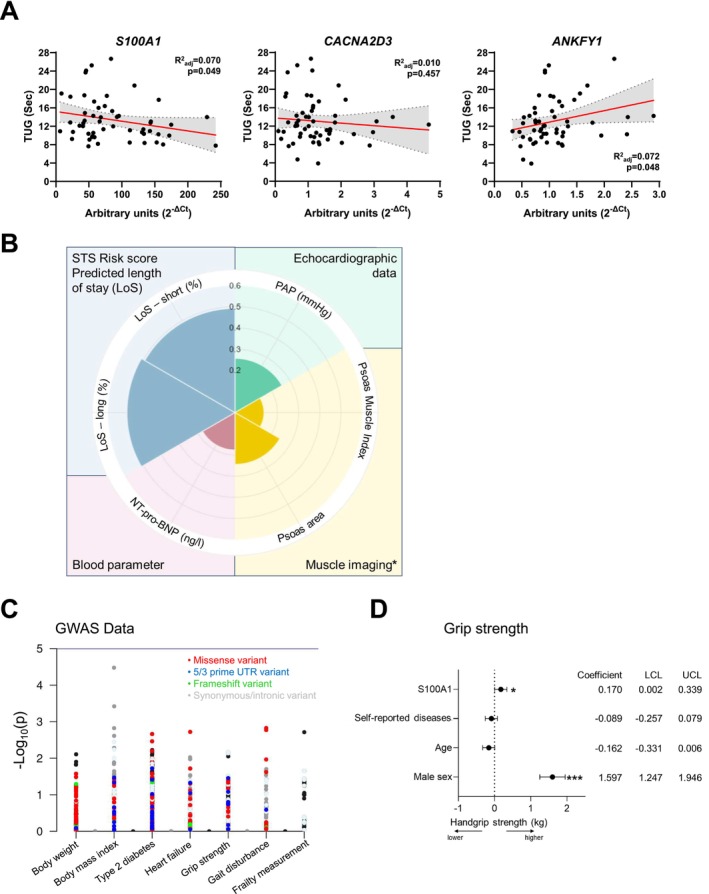
S100A1, a novel determinant of physical frailty. (A) qRT‐PCR results of *S100A1*, *CACNA2D3* and *ANKFY1* gene expression in human skeletal muscle biopsies and correlation to impaired mobility (TUG). (B) Spider plot representing the linear regression coefficient for different phenotypical parameters with a significantly altered *S100A1* gene expression pattern in muscle measured within the study cohort. (C) Manhattan plot for different traits associated with nucleotide variants within the *S100A1* gene ± 5 kb. (D) Linear regression of grip strength with different standardized variables adjusted for number of comorbidities, age and sex (*n* = 45). The graphs show the lower (LCL) and upper (UCL) confidence limit of 95% confidence intervals. *** *p* < 0.001, ** *p* < 0.01, * *p* < 0.05.

In order to identify genes encoding for secreted proteins, which may affect muscle and whole‐body metabolism on the level of organ crosstalk and can serve as possible blood biomarkers, the 10 overlapping genes between all physical frailty phenotypes were analysed via the Vertebrate Secretome Database (VerSeDa) [21] and the Human Protein Atlas (HPA). According to these data, *ANKFY1* (ankyrin repeat and FYVE domain containing 1), *CACNA2D3* (calcium voltage‐gated channel auxiliary subunit alpha2delta 3) and *S100A1* (S100 calcium binding protein A1) resemble secreted proteins, and therefore, we focused in the subsequent analysis on these genes and first validated their differential expression by qRT‐PCR. As shown in Figure 5A, *S100A1* revealed a significantly decreased expression with increasing time needed for the TUG test, whereas *ANFY1* expression was positively correlated. Moreover, the whole‐genome transcriptome data and the qRT‐PCR results clearly indicated that *S100A1* showed by far the highest expression level of all top 10 overlapping genes, making this gene an interesting candidate regulating physical frailty.

### 3.6 S100A1, a Novel Determinant of Physical Frailty

To figure out, whether *S100A1* gene expression is also associated with other phenotypical traits, the linear regression model or a group‐wise comparison was applied for all parameters measured. As already listed in the comparative analysis of differently expressed genes linked to physical frailty, *S100A1* expression was inversely correlated with a low gait speed and positively associated with higher handgrip strength (Figure 4B). Moreover, different parameters including muscle imaging (psoas muscle index and psoas area), echocardiographical data (PAP), metabolic traits (NT‐pro‐BNP) and risk assessment scores (e.g., STS short and long predicted LoS) were also changed with an altered *S100A1* expression (Figure 5B).

To further assess the impact of *S100A1* on muscle function and performance, we retrieved data from the human GWAS catalogue about the association of nucleotide variants located within the *S100A1* gene and 5‐kb upstream or downstream and traits related to frailty in other cohorts. Although no genome‐wide significant associations were found, several nucleotide variants showed correlations with grip strength, gait disturbance and frailty measurements. These correlations are illustrated in the Manhattan plot in Figure 5C, which displays single nucleotide polymorphisms (SNPs) along with their corresponding *p* values.

Moreover, to validate our findings in an independent cohort and to analyse whether S100A1 could serve as relevant biomarker for frailty, the S100A1 levels were measured in blood samples of a subset group of a study described elsewhere [S4] (main cohort characteristics are provided in the Table S1). In brief, this subcohort included 45 healthy community‐dwelling older adults (64.4% female; mean age 72.7 ± 5.9 years; 3 ± 2 self‐reported diseases), whereby individuals with cognitive impairments who were unable to understand spoken or written German and/or had severe neurodegenerative diseases (e.g., amyotrophic lateral sclerosis, Huntington's disease) were excluded. We observed that higher concentrations of S100A1 in plasma (mean 43.86 ± 216.74 ng/mL) were significantly correlated with higher handgrip strength of the participants (mean 33.1 ± 10.6 kg) measured using a hand dynamometer (Figure 5D).

**FIGURE 6:**
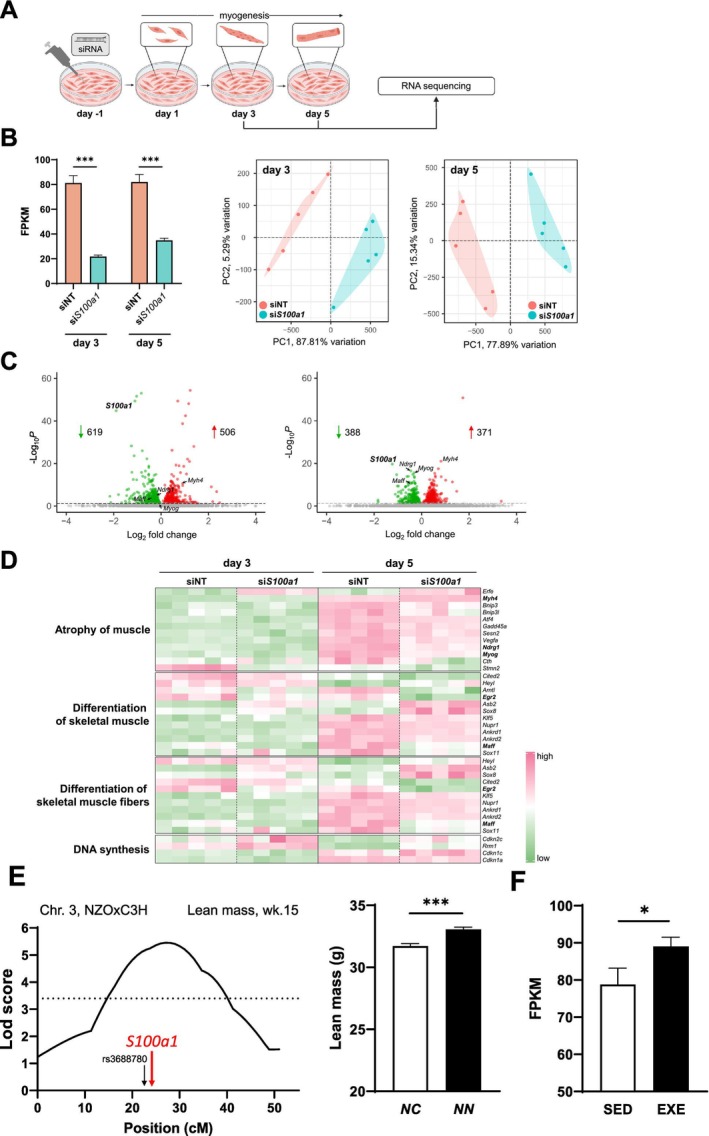
Impact of S100a1 on muscle function. (A) Transfection of C2C12 myoblasts with siRNA targeting *S100a1* one day before start of differentiation, and harvesting at Days 3 and 5. (B) Expression of *S100a1* in C2C12 cells after siRNA‐mediated knockdown in comparison to control cells treated with non‐targeting siRNA (left panel). Principal component analysis (PCA) of RNAseq data (*n* = 5/group) at Days 3 and 5 (right panel). (C) Volcano plots illustrating the differential gene expression between cells transfected with siRNA targeting *S100a1* and control cells. (D) Heat map displaying genes linked to significantly enriched pathways in si*S100a1* treated cells compared with controls at Day 5. Key regulators are highlighted in bold. (E) LOD score distribution of quantitative trait loci (QTL) derived from the NZOxC3H N2 population for lean mass at the age of 15 weeks and the corresponding values for the peak region of the QTL at rs3688780. Horizontal line represents genome‐wide significance. (F) *S100a1* expression in skeletal muscle (quadriceps) of C57BL/6 mice after 4 weeks of exercise training (*n* = 5). EXE, exercise; FPKM, fragments per kilobase per million mapped fragments; *NN*, homozygous NZO allele carriers; *NC*, heterozygous allele carriers; SED, sedentary controls. Data are presented as mean ± SEM, *** *p* < 0.001.

To explore the direct effects of S100A1 on muscle function, siRNA‐mediated knockdown of *S100Aa1* was performed in the myoblast cell line C2C12 prior induction of differentiation into myofibres. Cells were harvested for RNA sequencing on Days 3 and 5 post‐differentiation initiation, as depicted in Figure 6A. The RNAseq data showed a significant reduction in *S100a1* expression on Day 3 by 78% and on Day 5 by 65% (Figure 6B, left panel). Principal component analysis (PCA) performed subsequently at these time points revealed strong clustering dependent on the treatment group (Figure 6B, right panel). Overall, the response to the *S100a1* knockdown involved significant gene regulation changes: 619 genes were downregulated and 506 upregulated on Day 3, while 388 genes were downregulated and 371 upregulated on Day 5 (Figure 6C). To figure out whether these changes are linked to changes in skeletal muscle function, ingenuity pathway analysis (IPA) was performed and revealed four significant disease/functional annotations, atrophy of muscle, differentiation of skeletal muscle, differentiation of skeletal muscle fibres and DNA synthesis, when *S100Aa1* expression is reduced. Genes linked to these pathways included key regulators of skeletal muscle differentiation and proliferation such as *Myog* (myogenin) and *Maff* (MAF bZIP transcription factor F). As shown in the heat map in Figure 6D, most of these regulatory genes were decreased in gene expression at Day 5 after start of differentiation compared to cells treated with non‐targeting siRNA. These results underscore the vital role of S100A1 in skeletal muscle function and its potential implications in muscle frailty and related disorders.

Moreover, we also screened our previous studies in mice. Within the German Center for Diabetes Research (DZD), we initiated a cross project using four lean inbred mouse strains with varying T2D susceptibility, which were crossed with the obese and diabetes‐prone NZO mouse strain [22]. In the crossbreeding of NZO and C3H mice, one quantitative trait loci (QTL) for increased lean mass was identified on chromosome 3 (Figure 6E, left panel). Interestingly, the *S100a1* gene is located within the QTL peak region and was combined with an increased lean mass in homozygous NZO allele carriers (Figure 6E, right panel). Finally, screening of our transcriptome data of healthy C57BL/6J mice, which were subjected to a 4‐week exercise training (EXE) revealed a significantly higher *S100a1* expression in skeletal muscle after exercise compared with sedentary controls (SED, Figure 6F).

In conclusion, our data obtained in human and mouse studies position S100A1 as a novel regulator of skeletal muscle health and metabolism that warrants consideration of S100A1 as a potential indicator and target for the treatment of frailty in older cardiac patients.

## 4 Discussion

Frailty is a common and challenging condition in older cardiac patients, but still, it is difficult to determine the cause of frailty as the biological drivers of this multisystem dysregulation are manifold and likely to be interconnected [23]. Therefore, the current study aimed to identify the transcriptomic signature of frailty by screening older cardiac patients for different clinical parameters in combination with expression analysis in the respective skeletal muscle biopsies. More than two‐thirds of the patients exhibited one phenotype of physical frailty defined predominantly by impaired mobility, with a noticeable overlap with low gait speed and low strength. Noteworthy, alterations in expression of specific genes involved in physiological mechanisms of skeletal muscle were associated with phenotypes of frailty with similar overlapping patterns. Particularly, the calcium‐binding protein S100A1 exhibited a strong impact on muscle function and performance, which was strengthened by analysing data from functional tests in murine cells, different animal models as well as by human studies.

Physical frailty in our study cohort was primarily characterized by mobility limitations, as determined by the TUG test. This test focuses on functional mobility, assessed by measuring the time needed to complete a complex task of standing up from a chair, walking to a marker 3 m away, turning around the marker, walking back the 3 m and then sitting down again [S20]. Therefore, components of gait (e.g., acceleration, dynamic balance, turning and deceleration) and functional strength (get up and sit on a chair) are captured in this measurement [S21]. Poorer performance on the TUG correlates with reduced linear gait speed (5‐mWT) and low handgrip strength (index of overall strength) [24], [S22] which can explain the large overlap between frailty phenotypes in our cohort. Moreover, the TUG test was found to be suitable to characterize frail patients and to predict 1‐year mortality rate, for TAVI patients [25]. The trait of frailty identified in our TAVI cohort can be expected considering the older age of the patients and their higher predicted procedural risk for an adverse outcome. Consequently, complications were associated with impaired mobility in this vulnerable population.

Physical frailty has often been related to aspects of sarcopenia [10], which has been associated with a poorer prognosis, falls and lower quality of life in CVD patients [26] [S2]. One‐third of the included patients showed lower handgrip strength (< 27 kg male; < 16 kg female), which is one of the parameters that characterize sarcopenic patients [S2]. However, only one man showed a concomitant decrease in muscle mass, clinically confirming the condition of sarcopenia [26]. In our investigation the total body skeletal muscle mass of the patients was largely above the recommended cut‐off that defines sarcopenia [S2]. Pre‐interventional CT assessment can provide analysis of the patient's body composition and quantification of sarcopenia from a single axial image acquired at the level of the third lumbar vertebra [27] [S23]. It is thus possible to differentiate the relative proportions of various tissues including muscle, visceral and subcutaneous fat [28]. In the subgroup that underwent a CT, the quantification of psoas muscle area was also above a previously published indicator of frailty and sarcopenia [29]. Our results confirm that loss of muscle functionality interconnects phenotypes of frailty with sarcopenia in older CVD patients [13], whereby the determinants of sarcopenia alone could not elucidate the manifestation of frailty.

Several studies have already described the impact of genetics on frailty, but knowledge about the precise role of genetic factors is still fragmentary. Therefore, we have used a hypothesis‐free driven approach to identify novel factors implicated in disease progression by performing transcriptomics in frail patients. Overall, we have detected changes in different pathways known to regulate muscle metabolism and function with the highest number of genes to be dysregulated in correlation with an impaired gait speed. Among the top genes with an altered expression, the calcium‐binding protein *S100A1* was identified to be associated with a low gait speed, impaired mobility and low handgrip strength.

S100 proteins belong to a calcium‐binding cytosolic protein family, which composes of more than 20 known members [30]. S100 family members have a broad range of intracellular and extracellular functions that encompass the regulation of cell apoptosis, proliferation, differentiation, migration, energy metabolism, calcium balance, protein phosphorylation and inflammation [24, 30, 31, 32]. The S100 family member *S100z* was identified in our previous study as the most striking gene to be causal for the diabetes QTL *Nidd13/NZO* by affecting islet cell proliferation as well as apoptosis when overexpressed in mouse insulinoma (MIN6) cells [33].

In the current study, we have shown that S100A1 could play a pivotal role in the development of physical frailty. S100A1 has emerged as a central regulator of cardiomyocyte (CM) Ca^2+^ homeostasis and cardiac contraction [34] [S24, S25]. S100A1 expression is downregulated in heart failure with reduced ejection fraction (HFrEF) and right‐sided heart failure due to pulmonary hypertension, which contributes to progression and mortality in both diseases [35] [S26–S28]. Moreover, adenoviral delivery of S100A1 was effective in the treatment of heart failure in small and large animals and improving the failing function of animal and human cardiomyocytes, clearly positions S100A1 as an appropriate target for cardiac gene therapy [36] [S28, S29]. There are several pieces of evidence showing that S100A1 is also involved in fine‐tuning skeletal muscle Ca^2+^ release. S100A1 binds to ryanodine receptor type‐1 and plays an imperative role in the excitation‐contraction coupling (ECC) process of skeletal muscle [37], [S30] whereas Chaturvedi et al. found a decreased *S100A1* expression with time during the process of myogenesis [38]. A genetic overlap of frailty with CVD and its risk factors has already been reported, focusing on single nucleotide polymorphisms in GWAS. These studies have shown that frailty is associated with common genetic polymorphisms, many of which are implicated in CVD, supporting the hypothesis of a shared/common pathophysiology between the entities, which may also apply to *S100A1* as a multiple‐organ regulator.

To further asses the causal link between *S100A1* expression and muscle metabolism, functional assays were performed in the myoblast cell line C2C12. The results revealed profound changes in gene expression that coincide with critical pathways associated with muscle physiology and disease impacted by reduced *S100a1* expression, implicating this in both muscle maintenance and development. Genes such as the transcription factors *Myog* and *Maff*, which are critical regulators of muscle differentiation and growth, were notably affected. Myog (myogenin) is a member of the MyoD family of transcription factors and has pivotal roles during later stages of myogenesis, primarily influencing the terminal differentiation and functional maturation of myoblasts into myotubes and mature muscle fibres. Depletion of myogenin has been linked to severe muscle dystrophy and impaired muscle regeneration [S31, S32]. Similarly, *Myh4*, encoding myosin heavy chain (MHC) IIb, is a highly abundant contractile protein in muscle, and generally used as a marker for the maturation of C2C12 myofibres [S33]. Mice lacking Myh4 showed muscle weakness with reduced skeletal muscle mass [S34]. These examples suggest that S100A1 could be a central node in the regulatory network that governs muscle phenotype and functional capacity.

Next to the strong correlation of the *S100A1* gene expression with distinct parameters of physical frailty, we observed increased protein levels in plasma samples of older, healthy adults with higher handgrip strength. The data of this second independent cohort clearly validated the association between S100A1 and handgrip strength and furthermore the possibility of using S100A1 levels as potential blood biomarker for impaired muscle strength. Nonetheless, additional research is required on individuals with higher frailty status to ascertain the extent to which S100A1 can serve as a reliable and valid blood biomarker, and to what degree initial alterations in S100A1 expression indicate the early signs of altered frailty status. This research could potentially enable the early detection of changes in muscle function through S100A1 measurement, thereby facilitating early intervention to mitigate these developments.

Preventing and reversing frailty requires an integrative approach, in which physical activity, nutritional interventions, cognitive training and possibly a pharmacological approach may be included [39, 40]. Among these therapeutic interventions, physical exercise seems to be the most effective and popular one [13]. There is no evidence available in the literature to pinpoint which kind of exercise is more effective for frail individuals [41], but it seems that a multicomponent exercise intervention could be the best choice [42]. Such a multicomponent exercise programme is defined as a combined programme of endurance, strength, coordination, balance and flexibility exercises that have the potential to impact a variety of functional performance measures [13]. Interestingly, our data obtained from a 4‐week exercise training in C57BL/6J mice showed an increased *S100a1* expression compared with the sedentary control group.

As already stated, the underlying biological mechanisms contributing to the physical phenotypes of frailty are complex and multifactorial and accordingly, several genes besides *S100A1* have been detected with an altered expression and should therefore also be considered as determining factors in disease progression. For instance, the *CACNA2D3* gene, which encodes a member of the alpha‐2/delta subunit family, a protein in the voltage‐dependent calcium channel complex. *CACNA2D3* has been found to possess a potential tumor suppressor function in multiple carcinomas [S35] and loss‐of‐function mutations have been identified as risk factors for autism spectrum disorders (ASDs) in humans [S36–S39]. It was also reported that exogenous expression of *CACNA2D3* can strongly inhibit cell growth, migration, invasion and induce apoptosis [S40]. The protein encoded by the gene *SLC35D1* (*Solute carrier family 35 member D1*) resides in the endoplasmic reticulum (ER), which transports both UDP‐glucuronic acid (UDP‐GlcA) and UDP‐N‐acetylgalactosamine (UDP‐GalNAc) from the cytoplasm to the ER lumen [S41]. The *Slc35d1*‐deficient mouse showed a lethal form of skeletal dysplasia and human *SLC35D1* gene pathogenic variants have been described to cause autosomal recessive Schneckenbecken dysplasia [S42]. Another interesting candidate is *MYO19* (*Myosin‐19*), a gene considered to play a role in actin‐based mitochondria dynamics in cells, as it is involved in mitochondrial partitioning, [S43, S44] regulating the balance of fission and fusion, [S45] as well as being recently implicated in cristae organization [S46].

The present study has certain limitations. This investigation was conceptualized as an exploratory study to be conducted in a single centre for logistical reason, such as muscle specimens' collection and storage, as well as to simplify the integration of functional assessments into routine clinical practice. The associations of different phenotypes of frailty with periprocedural complications could not have been estimated a priori on the basis of a power analysis. This was due to consistent heterogeneity of studies assessing frailty in older patients with CVD (heterogeneity in age, interventions or surgical procedures and severity of disease) [S47]. Different components of frailty have been defined according to different assessments, and controversial effects on mortality, morbidity and major events have been presented, while a general consensus on the operationalisation of frailty was lacking during the study period [2]. Due to the tightly planned preoperative clinical procedures, it was not always possible to assess patients according to the standardized BIA measurement procedure (e.g., measurement done at different time of the day, control for fluid intake not always possible, > 5 h from last meal). However, it was demonstrated that fasting status and fluid intake did not affect the validity of the BIA measurements [S48].

In conclusion, our findings show that older CVD patients undergoing surgery or TAVI are characterized by preoperative phenotypes of physical frailty, primarily by limited mobility with concomitant low gait speed and reduced strength. Transcriptome analysis of skeletal muscle biopsies identified alterations in gene expression related to muscle metabolism and function that are associated with the physical determinants of frailty. Notably, among nine other candidates, the calcium‐binding protein *S100A1* emerged as a novel determinant of physical frailty. However, further studies, including functional assays and validation in an independent cohort, are needed to confirm the role of *S100A1* as a potential target for physical frailty in this population. Understanding the expression profile of frailty could provide valuable insights into preventive strategies to improve postoperative functioning‐related outcomes. Moreover, the study highlights the importance of considering multiple factors in assessing and addressing frailty in older populations, emphasizing the need for integrated, multicomponent interventions that encompass various aspects of functional health.

## Conflicts of Interest

The authors declare no conflicts of interest.

## Supporting information


**Table S1.** Participants characteristics: *n* = 45 healthy community‐dwelling.
**Table S2.** Participants characteristics: CVD subtype.
**Figure S1.** Summary of adjusted (age, sex, total number of comorbidities, estimated glomerular filtration rate) regression analyses (six models: A to E) that identified association between frailty phenotypes and perioperative complications in surgery and TAVI patients. OR: odds ratio; CI: confidence interval; TUG: Timed Up and Go test; 5‐mWT: 5‐Meter Walk Test; MNA‐sf: Mini Nutritional Assessment‐short form; MMSE: Mini‐Mental State Examination; TAVI: transcatheter aortic valve implantation.

## Data Availability

The datasets generated in the current study are available from the GEO database under GEO accession number: GSE287726.
