# Supplementary material for: Transcriptomic Signature of Frailty in Older Patients With Cardiovascular Disease Undergoing Cardiac Surgery or TAVI
Source: J Cachexia Sarcopenia Muscle. 2025 Jun 5;16(3):e13846. doi: 10.1002/jcsm.13846 (PMC12138287; doi:10.1002/jcsm.13846)
Supplement: Supplementary file 1 — Table S1. Participants characteristics: n = 45 healthy community‐dwelling. Table S2. Participants characteristics: CVD subtype. Figure S1. Summary of adjusted (age, sex, total number of comorbidities, estimated glomerular filtration rate) regression analyses (six models: A to E) that identified association between frailty phenotypes and perioperative complications in surgery and TAVI patients. OR: odds ratio; CI: confidence interval; TUG: Timed Up and Go test; 5‐mWT: 5‐Meter Walk Test; MNA‐sf: Mini Nutritional Assessment‐short form; MMSE: Mini‐Mental State Examination; TAVI: transcatheter aortic valve implantation. [file JCSM-16-e13846-s001.docx]

**Transcriptomic signature of frailty in older patients with cardiovascular disease** **undergoing cardiac surgery or TAVI**

Omar Baritello^1,2^, Simon H. Sündermann^3,4,5^, Kristian Espinosa-Garnica^3^, Jörg Kempfert^3,4,5^, Markus Jähnert^6,7^, Nick L. Beetz^5,8,9^, Dominik Geisel^8^, Jasmin Gaugel^2,6,10^, Julia Rominger^2,6,10^, Ursula Müller-Werdan^11,12^, Catrin Herpich^11,13,14^, Kristina Norman^11,13,14,15^, Alexandra Chadt^6,16^, Hadi Al-Hasani^6,16^, Heinz Völler^1^, Annett Salzwedel^1^ & Heike Vogel^2,6,7,10*^

1) Department of Rehabilitation Medicine, Faculty of Health Sciences Brandenburg, University of Potsdam, Potsdam, Germany

2) Research Group Molecular and Clinical Life Science of Metabolic Diseases, Faculty of Health Sciences Brandenburg, University of Potsdam, Potsdam, Germany

3) Department of Cardiothoracic and Vascular Surgery, Deutsches Herzzentrum der Charité, Berlin, Germany

4) Charité- Universitätsmedizin Berlin, Berlin, Germany

5) German Center for Cardiovascular Research (DZHK), Partner Site Berlin, Berlin, Germany

6) German Center for Diabetes Research (DZD e.V.), München-Neuherberg, Germany

7) Department of Experimental Diabetology, German Institute of Human Nutrition Potsdam-Rehbruecke, Nuthetal, Germany

8) Department of Radiology, Charité-Universitätsmedizin Berlin, Corporate Member of Freie Universität Berlin and Humboldt-Universität zu Berlin, Berlin, Germany

9) Berlin Institute of Health at Charité –Universitätsmedizin Berlin, BIH Biomedical Innovation Academy, Berlin, Germany Berlin Institute of Health, Berlin, Germany

10) Research Group Nutrigenomics of Obesity, German Institute of Human Nutrition Potsdam-Rehbruecke, Nuthetal, Germany

11) Department of Geriatrics and Medical Gerontology, Charité-Universitätsmedizin Berlin, Corporate Member of Freie Universität Berlin and Humboldt-Universität zu Berlin, 10117, Berlin, Germany

12) Evangelisches Geriatriezentrum Berlin gGmbH, 13347, Berlin, Germany

13) Department of Nutrition and Gerontology, German Institute of Human Nutrition Potsdam-Rehbrücke, 14558 Nuthetal, Germany

14) Institute of Nutritional Science, University of Potsdam, 14558 Nuthetal, Germany

15) German Center for Cardiovascular Research (DZHK), Partner Site Berlin, 10785 Berlin, Germany

16) Institute for Clinical Biochemistry and Pathobiochemistry, German Diabetes Center (DDZ), Medical Faculty, Heinrich Heine University, Duesseldorf, Germany

*OB and SS share first authorship of this manuscript*

*AS and HV share senior authorship of this manuscript*

***Corresponding author:**

Heike Vogel, PhD

E-mail: heikevogel@dife.de

Tel: +49 33200 88 4545

Fax: +49 33200 88 2334

**1. Materials and Methods**

- 1. **Study setting and patients**

Exclusion criteria were age < 70 years, severe cognitive impairment (e.g. dementia), clinical instability (e.g. instable vital signs), dialysis, liver cirrhosis, severe chronic obstructive pulmonary disease (GOLD III-IV), cancer (under treatment or life expectancy < 1 year), need for oxygen, emergent surgery/intervention, language barrier or patient withdrawal. All included participants gave written informed consent.

- 1. Sample size justification was based on a power analysis for genome-wide gene expression analysis, performed using *G*Power* (version 3.1.9.7), assuming a simple linear regression model testing the deviation of R^2^ from zero, a medium effect size (Cohen’s f^2^=0.15), a 5% false discovery rate (FDR) controlled by the Benjamini-Hochberg procedure and a target power of 80%.**Body composition and muscle imaging**

For analysis of body composition an automated artificial intelligence-based image segmentation tool was used. The software is integrated in the commercially available picture archiving and communication system (PACS) Visage version 7.1. (Visage Imaging GmbH). It is based on a convolutional neural network, U-net, and has been internally and externally validated. [S13] The areas (in cm^2^) and densities (Hounsfield units) of the different tissue classes including psoas muscle and skeletal muscle at the third lumbar vertebra are automatically calculated. As in this study patients underwent CT scans with a field of view smaller than the complete abdomen, we analysed the body composition parameters psoas muscle area (PA) and psoas muscle index (PMI), as the psoas muscle was always completely imaged within the field of view of the CT scan. The psoas skeletal muscle index (PMI) was calculated using the following formula: psoas muscle area (cm^2^) / body surface area (m^2^).

- 1. **Functional assessments**

The assessment procedures were defined in advance and followed a standardised protocol. All measurements were performed by the same trained scientist on all included patients.

**5-meter Walk Test (5-mWT)** [S1]

The 5-mWT was used to assess gait speed. The patients were instructed to start walking on a start signal “at their usual gait speed” in a straight line for a distance of 7 meters, which was defined by two cones. The first and the final one meter of the course (marked with coloured tape on the floor) served as acceleration and deceleration phases, respectively. Only the time (sec) needed to walk the central 5 meters of the course was measured using a stopwatch. Two measurements were performed with 30 sec recover in between and the mean value of the measurements was calculated. Walking aids such as stick or a rollator were allowed. Based on the mean value, the patients were classified into: “unable to perform” (patient is unable to/doesn’t want perform the test), “slow gait speed” (≥ 6 sec) or “robust” (< 6 sec).

**Handgrip strength (hand-dynamometer)** [S2]

The maximal voluntary isometric handgrip strength (kg) was measured with a digital hand dynamometer (FR-Jamar® Plus; Performance Health, UK). The dominant hand of the patients was assessed in a seated position with the shoulder adducted and neutrally rotated. The elbow was flexed to 90° with forearm and wrist in a neutral position. On the start signal, the participant grasped the handle of the dynamometer to the maximal extent (maximal voluntary isometric contraction) until the stop signal (3 sec). Two measurements were performed, with 30 seconds recover in between, and the mean value of the measurements was calculated. Patients were classified based on the mean value as: “unable to perform” (patient is unable to/doesn’t want to perform the test), “weak” (< 27 kg male; < 16 kg female) or “robust” (≥ 27 kg male; ≥ 16 kg female) as recommended by the EWGSOP2.

**Timed Up-and-Go test (TUG)** [S3]

The test procedure consisted on getting up from a chair, walking to a marker set at the distance of 3 meters, turn around the marker, walk the 3 meters back and then sit down again. Patients were instructed to perform the task on start signal at their usual walking speed and the time (seconds) required to complete the assignment was recorded using a stopwatch. Walking aids such as cane or a rollator were allowed, if necessary. Two measurements were performed with one-minute recover in between, and the mean value of the measurements was calculated. Based on the mean value the patients were stratified as: “unable to perform” (patient is unable to/doesn’t want to perform the test), “severely impaired mobility” (≥ 20 sec), “moderately impaired mobility” (≥ 10 to < 20 sec) or “robust” (< 20 sec).

- 1. **Measurement of S100A1 in serum samples**

To validate our results in a similar cohort we used a subset of a study described elsewhere [S4]. In brief, healthy community-dwelling, older adults were recruited via internet and flyers during the time period 03/2017 to 06/2017. Inclusion criterion was age between 60 and 85 years. Exclusion criteria were cognitive impairment, inability to understand verbal or written German as well as severe neurodegenerative diseases (e.g. amyotrophic lateral sclerosis, Huntington’s disease). The study was approved by the ethics committee of the Charité - Universitätsmedizin Berlin and was registered at clinicaltrials.gov as NCT02994901. All participants signed a written informed consent. Comorbidities were self-reported and not assessed systematically (2. Supplementary Table). Maximum hand grip strength was measured using the JAMAR hand dynamometer (Preston Bissell Health Care Co., Jackson, MI, USA), the highest value of three attempts was recorded. Blood was drawn between 7 and 8 am after an overnight fast. Serum was obtained after 30 min incubation and centrifugation at 2,250 g for 15 min. Serum samples were stored at -80°C until further analysis. S100A1 concentrations were determined by an ELISA assay according to manufacturer’s instructions using commercial kits (Thermo Fisher Scientific) with an assay sensitivity of 0.82 ng/ml.

- 1. **Cultivation and transfection of C2C12 cells**

Cells of the murine muscle cell line C2C12 were maintained in 75 cm^2^ cell culture flasks with 10 ml high glucose (4.5 g/l) Dulbecco’s Modified Eagle Medium (DMEM-HG, PAN-Biotech) supplemented with 10 % fetal calf serum (FCS, Gibco®, Carlsbad, USA) under standard conditions (37°C, 5 % CO2). Cells were passaged at 60-70 % confluence using trypsin. To establish knockdown of *S100a1* expression, C2C12 cells were treated with a siRNA mix targeted against *S100a1* (ON-TARGETplus mouse S100a1 siRNA SMARTpool, Horizon Discovery, no. L-041031-01-0010) in a final concentration of 100 nm. A non-targeting siRNA mix was used as control (ON-TARGETplus non-targeting pool, Horizon Discovery, no. D-001810-10-20). The siRNA was introduced into the cells by floating transfection, where cells were transfected while seeding. Therefore, the siRNA and the transfection reagent Lipofectamine 2000 (Invitrogen by Thermo Fisher Scientific) were diluted in serum-free high glucose (4.5 g/l) Dulbecco’s Modified Eagle Medium (DMEM-HG, PAN-Biotech) and incubated according to the manufacturer’s protocols. During 20 min incubation of siRNA and transfection reagent, cells were trypsinized, 55,000 cells per well were spun down for 5 min at 1,000 rpm and pellets were resuspended in the respective siRNA-Lipofectamine mix. After 15 min incubation at 37°C and 5 % CO2, 400 µl serum-free DMEM-HG per well were added to each suspension and subsequently 500 µl of cell suspension were seeded per well. After another 5 h, 500 µl DMEM-HG containing 20 % serum (FCS, Gibco®) were added to each well. Cell differentiation was induced 48 h after transfection by applying low-serum medium (DMEM-HG supplemented with 2 % horse serum). Cells were harvested 48 h, 96 h or 144 h after transfection.

- 1. **Gene expression analysis in skeletal muscle and C2C12 myotubes**

All RNA samples extracted from *m. quadriceps femoris* and C2C12 cells with RNA integrity number RIN ≥7 (Bioanalyser, Agilent Technologies, Germany), were selected for transcriptome analysis.

Whole-genome transcriptome analysis was done by RNA-sequencing, carried out by BGI Group (Yantian District, Shenzhen, China). The final library was generated using BGISEQ. BGI delivered raw data. After sequencing, adapters and low-quality reads were filtered and FastQC v0.11.8 was conducted to check quality of the samples. Next, reads were aligned to the reference genome (GRCh38/hg38) using STAR v.2.7.0f, and FPKM (fragments per kilobase per million) values for transcripts were determined by STRINGTIE v1.3.6, both with default options for paired reads. For regression analysis, a linear model using OLS (ordinary least squares); formula: phenotype ~ gene expression with bgi_batch, sex, age, filtration rate and comorbidity as cofactors was performed. Associations between gene expression and phenotype were considered as significant when p≤0.05. Standardized parameters were obtained by fitting the model on a standardized version of the dataset. Confidence intervals (CIs) of 95% and p-values were computed using the Wald approximation.

For C2C12 cells, whole-transcriptome was also performed by BGI including the alignment of quality-controlled reads to the GRCm39 reference (release 105) using Bowtie2(v2.2.5). Gene expression has been calculated via RSEM(v1.2.8) and group comparison has been performed via DESeq2(v1.38.3) pipeline. Network analysis was obtained with Ingenuity Pathway Analysis (IPA) (Qiagen).

Gene set enrichment for Kyoto Encyclopedia of Genes and Genomes (KEGG) pathways was analysed with Database for Annotation, Visualization and Integrated Discovery (DAVID) [S14] and pathway analysis. Regression, KEGG analysis was carried out using the R Statistical Software (v4.1.2; R Core Team 2021).

For quantitative real-time PCR (qRT-PCR) total RNA extracted from *m. quadriceps femoris* was reversed transcribed (M-MVL RT, Promega, Madison, WI, USA). Genes of interest were detected using specific IDT (Integrated DNA Technologies, Coralville, IA, USA) probes (S100A1: Hs.PT.58.19383908; CACNA2D3: Hs.PT.58.1713690; ANKFY1: Hs.PT.58.40561587). Expression levels were evaluated using the 2(-Delta C(T)) method [S49] with β-actin (ACTB: Hs.PT.39a.22214847) as internal control.

**2. Supplementary Table**

| **Supplementary Table 1. Participants characteristics: n = 45 healthy community-dwelling** | | |
| --- | --- | --- |
| **Variable** | **Mean ± SD**  **[Median (25_th_; 75_th_)]**  **n (%)** | **Range Min - Max** |
| Age (years) | 72.7 ± 5.9 | 61.7 - 83.2 |
| Sex (% female) | 64.4 |  |
| Self-reported diseases (n) | 3 ± 2 [2 (1; 4)] | 0 - 9 |
| CVD | 31 (79.5) |  |
| Orthopaedic | 19 (48.7) |  |
| High cholesterol | 11 (28.2) |  |
| Diabetes | 4 (10.3) |  |
| Grip strength (kg) | 33.1 ± 10.6 | 16.5 - 64.0 |
| SD: standard deviation; Median (25th; 75th): median with 25th to 75th interquartile; Range: minimum - maximum; CVD: cardiovascular diseases (e.g. hypertension, coronary heart diseases); Orthopaedic: e.g. osteoarthritis, osteoporosis | | |

**Supplementary Table 2. Participants characteristics: CVD subtype**

| **CVD subtype** | **Number of participants** |
| --- | --- |
| Aortic valve stenosis | 32 |
| Aortic valve stenosis and mitral valve stenosis | 1 |
| Mitral valve stenosis | 2 |
| Coronary artery disease (CAD) | 19 |
| CAD and aortic valve stenosis | 6 |
| CAD and atrial fibrillation | 3 |

CVD: cardiovascular diseases

**3. Supplementary Figure**

**
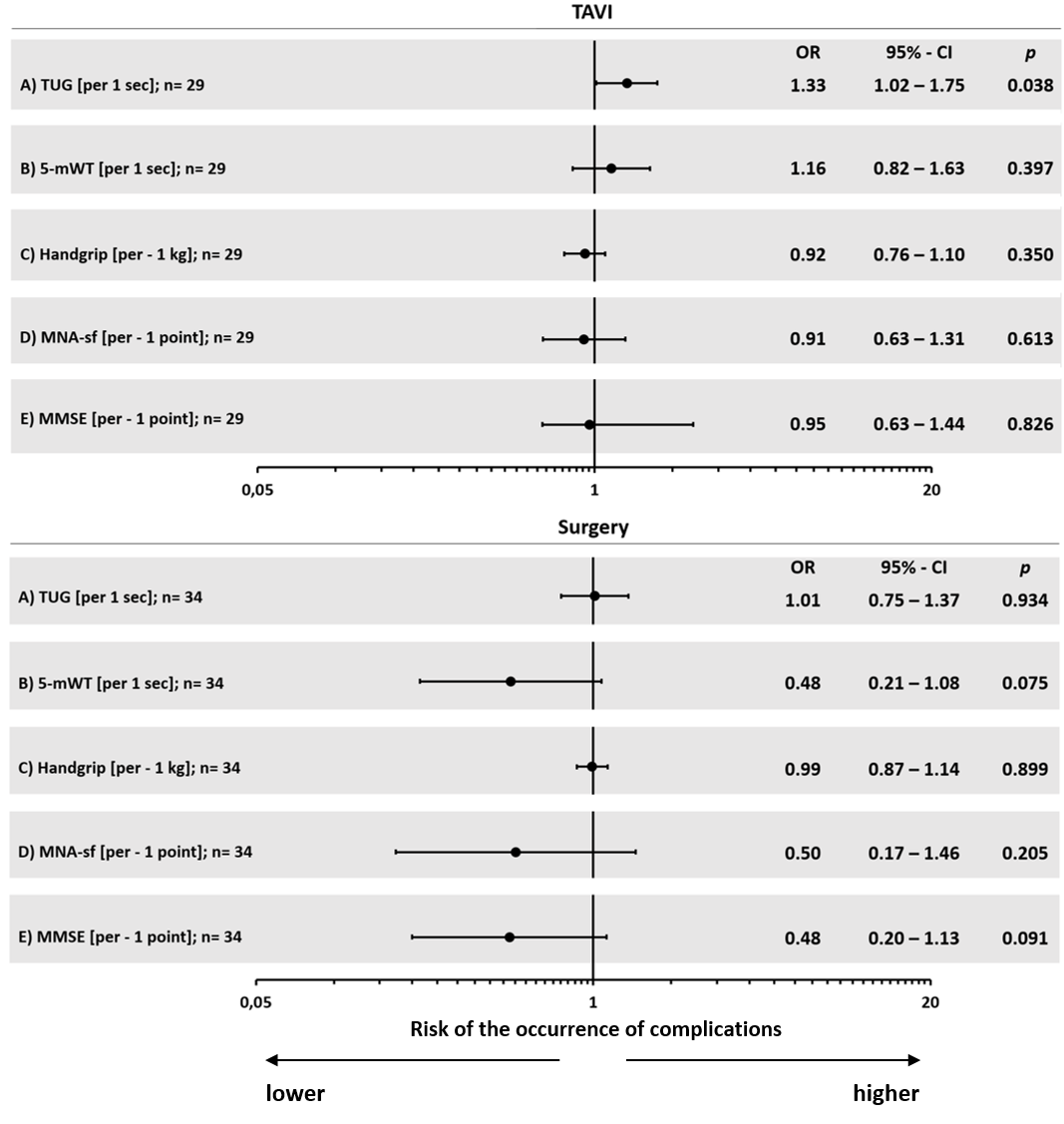
**

**Supplementary Figure 1.** Summary of adjusted (age, sex, total number of comorbidities, estimated glomerular filtration rate) regression analyses (six models: A to E) that identified association between frailty phenotypes and perioperative complications in surgery and TAVI patients. OR: odds ratio; CI: confidence interval; TUG: Timed Up-and-Go test; 5-mWT: 5-Meter Walk Test; MNA-sf: Mini Nutritional Assessment-short form; MMSE: Mini Mental State Examination; TAVI: transcatheter aortic valve implantation

**4. References**

S1. Afilalo J, Kim S, O’Brien S, Brennan JM, Edwards FH, Mack MJ et al. Gait speed and operative mortality in older adults following cardiac surgery. JAMA Cardiol 2016;1:314–321.

S2. Cruz-Jentoft AJ, Bahat G, Bauer J, Boirie Y, Bruyère O, Cederholm T et al. Sarcopenia: revised european consensus on definition and diagnosis. Age Ageing 2019;48:16–31.

S3. Baritello O, Salzwedel A, Sündermann SH, Niebauer J, Völler H. The pandora’s box of frailty assessments: which is the best for clinical purposes in TAVI patients? A critical review. J Clin Med 2021;10.

S4. Herpich C, Franz K, Ost M, Otten L, Coleman V, Klaus S, Müller-Werdan U, Norman K. Associations between serum GDF15 concentrations, muscle mass, and strength show sex-specific differences in older hospital patients. Rejuvenation Res 2021;24:14-19.

S5. Folstein MF, Folstein SE, McHugh PR. Mini-mental state. 1975;1975:189–198.

S6. Rubenstein LZ, Harker JO, Salvà A, Guigoz Y, Vellas B. Screening for undernutrition in geriatric practice: developing the short-form mini-nutritional assessment (MNA-SF). J Gerontol A Biol Sci Med Sci 2001;56:M366-72.

S7. Katz S, B. Ford A, W. Moskowitz R, A. Jackson B, W. Jaffe M. Studies of illness in the aged. JAMA 1963;Sept.

S8. Lawton MP, Brody EM. Assessment of older people: self-maintaining and instrumental activities of daily living. Gerontologist 1969;9:179–186.

S9. Ware J, Kosinski M, Keller SD. A 12-item short-form health survey: construction of scales and preliminary tests of reliability and validity. Med Care 1996;34:220–233.

S10. Martin A, Rief W, Klaiberg A, Braehler E. Validity of the brief patient health questionnaire mood scale (PHQ-9) in the general population. Gen Hosp Psychiatry 2006;28:71–77.

S11. Otten L, Stobäus N, Franz K, Genton L, Müller-Werdan U, Wirth R et al. Impact of sarcopenia on 1-year mortality in older patients with cancer. Age and aging 2019;2019:413–418.

S12. Shahian DM, Jacobs JP, Badhwar V, Kurlansky PA, Furnary AP, Cleveland JC et al. The society of thoracic surgeons 2018 adult cardiac surgery risk models: Part 1-background, design considerations, and model development. Ann Thorac Surg 2018;105:1411–1418.

S13. Kappetein AP, Head SJ, Généreux P, Piazza N, van Mieghem NM, Blackstone EH et al. Updated standardized endpoint definitions for transcatheter aortic valve implantation: the valve academic research consortium-2 consensus document. J Thorac Cardiovasc Surg 2013;145:6–23.

S14. Beetz NL, Maier C, Segger L, Shnayien S, Trippel TD, Lindow N et al. First PACS‐integrated artificial intelligence‐based software tool for rapid and fully automatic analysis of body composition from CT in clinical routine. JCSM Clin Rep 2022;7:3–11.

S15. Da Huang W, Sherman BT, Lempicki RA. Bioinformatics enrichment tools: paths toward the comprehensive functional analysis of large gene lists. Nucleic Acids Res 2009;37:1–13.

S16. Vogel H, Kamitz A, Hallahan N, Lebek S, Schallschmidt T, Jonas W et al. A collective diabetes cross in combination with a computational framework to dissect the genetics of human obesity and Type 2 diabetes. Hum Mol Genet 2018;27:3099–3112.

S17. Broman KW, Wu H, Sen S, Churchill GA. R/qtl: QTL mapping in experimental crosses. Bioinformatics 2003;19:889–890.

S18. Kanzleiter T, Jähnert M, Schulze G, Selbig J, Hallahan N, Schwenk RW et al. Exercise training alters DNA methylation patterns in genes related to muscle growth and differentiation in mice. Am J Physiol Endocrinol Metab 2015;308:E912-20.

S19. Sollis E, Mosaku A, Abid A, Buniello A, Cerezo M, Gil L et al. The NHGRI-EBI GWAS Catalog: knowledgebase and deposition resource. Nucleic Acids Res 2023;51:D977–D985.

S20. Podsiadlo D, Richardson S. The timed up go: A test of basic functional mobility for frail elderly persons. JAGS 1991;1991:142–148.

S21. Herman T, Giladi N, Hausdorff JM. Properties of the ‘timed up and go’ test: more than meets the eye. Gerontology 2011;57:203–210.

S22. Alonso AC, Ribeiro SM, Luna NMS, Peterson MD, Bocalini DS, Serra MM et al. Association between handgrip strength, balance, and knee flexion/extension strength in older adults. PLoS One 2018;13:e0198185.

S23. Gibson DJ, Burden ST, Strauss BJ, Todd C, Lal S. The role of computed tomography in evaluating body composition and the influence of reduced muscle mass on clinical outcome in abdominal malignancy: a systematic review. Eur J Clin Nutr 2015;69:1079–1086.

S24. Rohde D, Busch M, Volkert A, Ritterhoff J, Katus HA, Peppel K et al. Cardiomyocytes, endothelial cells and cardiac fibroblasts: S100A1’s triple action in cardiovascular pathophysiology. Future Cardiol 2015;11:309–321.

S25. Rohde D, Ritterhoff J, Voelkers M, Katus HA, Parker TG, Most P. S100A1: a multifaceted therapeutic target in cardiovascular disease. J Cardiovasc Transl Res 2010;3:525–537.

S26. Most P, Seifert H, Gao E, Funakoshi H, Völkers M, Heierhorst J et al. Cardiac S100A1 protein levels determine contractile performance and propensity toward heart failure after myocardial infarction. Circulation 2006;114:1258–1268.

S27. Pleger ST, Shan C, Ksienzyk J, Bekeredjian R, Boekstegers P, Hinkel R et al. Cardiac AAV9-S100A1 gene therapy rescues post-ischemic heart failure in a preclinical large animal model. Sci Transl Med 2011;3:92ra64.

S28. Brinks H, Rohde D, Voelkers M, Qiu G, Pleger ST, Herzog N et al. S100A1 genetically targeted therapy reverses dysfunction of human failing cardiomyocytes. J Am Coll Cardiol 2011;58:966–973.

S29. Pleger ST, Most P, Boucher M, Soltys S, Chuprun JK, Pleger W et al. Stable myocardial-specific AAV6-S100A1 gene therapy results in chronic functional heart failure rescue. Circulation 2007;115:2506–2515.

S30. Prosser BL, Wright NT, Hernãndez-Ochoa EO, Varney KM, Liu Y, Olojo RO et al. S100A1 binds to the calmodulin-binding site of ryanodine receptor and modulates skeletal muscle excitation-contraction coupling. J Biol Chem 2008;283:5046–5057.

S31. Hasty P, Bradley A, Morris JH, Edmondson DG, Venuti JM, Olson EN, Klein WH. Muscle deficiency and neonatal death in mice with a targeted mutation in the myogenin gene. Nature. 1993;364(6437):501-6.

S32. Nabeshima Y, Hanaoka K, Hayasaka M, Esumi E, Li S, Nonaka I, Nabeshima Y. Myogenin gene disruption results in perinatal lethality because of severe muscle defect. Nature. 1993 Aug 5;364(6437):532-5.

S33. Brown DM, Parr T, Brameld JM. Myosin heavy chain mRNA isoforms are expressed in two distinct cohorts during C2C12 myogenesis. J Muscle Res Cell Motil. 2012 Mar;32(6):383-90.

S34. Acakpo-Satchivi LJ, Edelmann W, Sartorius C, Lu BD, Wahr PA, Watkins SC, Metzger JM, Leinwand L, Kucherlapati R. Growth and muscle defects in mice lacking adult myosin heavy chain genes. J Cell Biol. 1997 Dec 1;139(5):1219-29.

S35. Nie C, Qin X, Li X, Tian B, Zhao Y, Jin Y et al. CACNA2D3 enhances the chemosensitivity of esophageal squamous cell carcinoma to cisplatin via inducing Ca2+-mediated apoptosis and suppressing PI3K/Akt pathways. Front Oncol 2019;9:185.

S36. Iossifov I, Ronemus M, Levy D, Wang Z, Hakker I, Rosenbaum J et al. De novo gene disruptions in children on the autistic spectrum. Neuron 2012;74:285–299.

S37. Girirajan S, Dennis MY, Baker C, Malig M, Coe BP, Campbell CD et al. Refinement and discovery of new hotspots of copy-number variation associated with autism spectrum disorder. Am J Hum Genet 2013;92:221–237.

S38. Rubeis S, He X, Goldberg AP, Poultney CS, Samocha K, Cicek AE et al. Synaptic, transcriptional and chromatin genes disrupted in autism. Nature 2014;515:209–215.

S39. Bracic G, Hegmann K, Engel J, Kurt S. Impaired subcortical processing of amplitude-modulated tones in mice deficient for Cacna2d3, a risk gene for autism spectrum disorders in humans. eNeuro 2022;9.

S40. Li Y, Zhu C-L, Nie C-J, Li J-C, Zeng T, Zhou J et al. Investigation of tumor suppressing function of CACNA2D3 in esophageal squamous cell carcinoma. PLoS One 2013;8:e60027.

S41. Hadley B, Litfin T, Day CJ, Haselhorst T, Zhou Y, Tiralongo J. Nucleotide sugar transporter SLC35 family structure and function. Comput Struct Biotechnol J 2019;17:1123–1134.

S42. Hiraoka S, Furuichi T, Nishimura G, Shibata S, Yanagishita M, Rimoin DL et al. Nucleotide-sugar transporter SLC35D1 is critical to chondroitin sulfate synthesis in cartilage and skeletal development in mouse and human. Nat Med 2007;13:1363–1367.

S43. Moore AS, Coscia SM, Simpson CL, Ortega FE, Wait EC, Heddleston JM et al. Actin cables and comet tails organize mitochondrial networks in mitosis. Nature 2021;591:659–664.

S44. Rohn JL, Patel J V, Neumann B, Bulkescher J, Mchedlishvili N, McMullan RC et al. Myo19 ensures symmetric partitioning of mitochondria and coupling of mitochondrial segregation to cell division. Curr Biol 2014;24:2598–2605.

S45. Majstrowicz K, Honnert U, Nikolaus P, Schwarz V, Oeding SJ, Hemkemeyer SA et al. Coordination of mitochondrial and cellular dynamics by the actin-based motor Myo19. J Cell Sci 2021;134.

S46. Shi P, Ren X, Meng J, Kang C, Wu Y, Rong Y et al. Mechanical instability generated by myosin 19 contributes to mitochondria cristae architecture and OXPHOS. Nat Commun 2022;13:2673.

S47. Veronese N. Frailty and cardiovascular diseases. Springer International Publishing: Cham; 2020.

S48. Artero A, Sáez Ramírez T, Muresan BT, Ruiz-Berjaga Y, Jiménez-Portilla A, Sánchez-Juan CJ. The effect of fasting on body composition Assessment in Hospitalized Cancer Patients. Nutr Cancer 2023;1–9.

S49. Livak KJ, Schmittgen TD. Analysis of relative gene expression data using real-time quantitative PCR and the 2(-Delta Delta C(T)) Method. Methods. 2001;402-8.
